# Geoenvironmental and Health Indices to Assess the Hazardousness of Heavy Metals in Urban Dust in Schoolyards in Murcia, Spain

**DOI:** 10.3390/toxics12110804

**Published:** 2024-11-07

**Authors:** María José Delgado-Iniesta, Pura Marín-Sanleandro, María del Carmen Canca Pedraza, Elvira Díaz-Pereira, Antonio Sánchez-Navarro

**Affiliations:** 1 Department of Agricultural Chemistry, Geology and Pedology, Faculty of Chemistry, Campus de Espinardo, University of Murcia, 30100 Murcia, Spain; delini@um.es (M.J.D.-I.); mcarmen.canca@um.es (M.d.C.C.P.); antsanav@um.es (A.S.-N.); 2 Soil and Water Conservation Research Group, Spanish National Research Council (CEBAS-CSIC), Campus de Espinardo, 30100 Murcia, Spain; ediazpereira@cebas.csic.es

**Keywords:** contamination, pollution, urban dust, heavy metals, geoenvironmental indices, risk indices, school playgrounds

## Abstract

The aim of this study was to evaluate the possible contamination of urban dust in the schoolyards of 27 schools in an urban area of the city of Murcia (SE Spain). The color and degree of magnetism, as well as the heavy metal content (Cd, Cu, Cr, Ni, Pb, and Zn), were determined to establish the absence or the degree of contamination, if present, using environmental and health indices. It was established that the concentrations of heavy metals in the dust samples followed the order Zn (454 mg kg^−1^) > Cu (77 mg kg^−1^) > Cr (68 mg kg^−1^) > Pb (56 mg kg^−1^) > Ni (19 mg kg^−1^) > Cd (0.4 mg kg^−1^). Dark-colored dust showed the highest concentrations of contaminants associated with medium or high magnetism. An analysis of the magnetic and non-magnetic fractions indicated the highest concentrations of all heavy metals in the magnetic fraction. According to the geoenvironmental indices used, the ecological risk in these schoolyards is moderate overall. Based on the health indices, ingestion is the main route of entry of dust particles into the body, which poses the main health risk for adults and children for all heavy metals. Regarding the hazard index (HI) for all elements and the cancer risk (CR) for children and adults, the results indicate that there is no health risk.

## 1. Introduction

Urban dust is a heterogeneous mixture of different elements and pollutant particles originating from natural and/or anthropogenic activities, such as erosion, industrial processes, heating, construction, and, above all, road traffic [1]. These particles, after dissipation into the atmosphere, are deposited on surfaces. Among the pollutants that make up urban dust, perhaps the most dangerous to health, are heavy metals due to their toxicity, posing various risks to humans, such as dermal and respiratory disorders and, above all, carcinogenic effects. Arsenic and cadmium cause carcinogenic effects in the body, affecting the skin, prostate, and bladder, among other organs [2]. The proximity of humans to sources of pollution in urban areas increases the risk of developing various diseases, with increasing mortality and morbidity rates [3]. In recent years, oncologists have been confronted with a growing phenomenon that they cannot fully explain: an increasing number of people are developing lung cancer without ever having smoked [4]. Contact with heavy metals contained in urban dust can occur in three different ways, namely, via direct oral ingestion of substrate particles, via inhalation of suspended particles through the mouth and nose, and via dermal absorption on exposed body parts [5]. The risk associated with the presence of toxic elements in urban dust has been extensively studied [6]. In addition, the smaller the particles, the more dangerous they are to the body, as they can reach the pulmonary alveoli. The most polluting types are *PM*_10_ (particles with a diameter of ≤10 μm) and *PM*_2.5_ (particles with a diameter of ≤2.5 μm), whose composition has been extensively studied [7,8]. Whereas *PM*_10_ are coarse particles derived mainly from combustion processes, *PM*_2.5_ are derived mostly from mechanical processes. Their presence has been studied in air-conditioning filters [9]. These particles are released into the environment, accumulate on different surfaces, and become part of urban dust. In each city, the concentration and size of these particles can vary depending on the climate, location, or their sources of origin. In a study by Linares and Diaz [10], it was found that particles with a diameter of less than 2.5 µm were closely related to hospital admissions of children under 10 years of age, as the concentrations of these particles exceeded the limit value set by the World Health Organization (WHO). Children are more vulnerable to these particles because their lungs are not yet fully developed, so if they suffer from respiratory diseases, these may be worsened by exposure to particulate matter. The WHO continually disseminates global air quality guidelines, the aim being that each state should seek to reduce pollutant emissions and reduce the adverse effects of these emissions on human health. In addition, toxic chemicals in the environment can cause neurodevelopmental disabilities, and the developing brain can be particularly sensitive to environmental pollutants [11]. As urban dust results from a mixture of different pollutants, attention should be paid not only to air quality guidelines but also to soil quality criteria and standards, as soil quality is also affected by dust. These standards are designed to regulate soil quality in accordance with the Spanish Royal Decree RD 9/2005 of 14 January 2005, which establishes the list of potentially soil-polluting activities and the criteria and standards for the declaration of contaminated soils [12] in order to protect public health. Urban soil is a major component of urban ecosystems, contributing directly or indirectly to health and well-being by supporting housing, schools, transport infrastructure, and leisure activities, filtering substances, and moderating the urban climate [13].

Due to their physiological and behavioral characteristics, children are more exposed to some environmental pollutants than adults. Since children are the most vulnerable group regarding health problems derived from urban pollution, it is important to carry out a study in schoolyards, places where children spend a large part of the school day, especially the youngest ones. It is in schoolyards where children can come into direct contact with urban dust by any of the routes described above (ingestion, inhalation, or dermal contact), especially in cities where the climate allows children to play outdoors all year round.

The color of urban dust is a characteristic that some authors have used to make a quick diagnosis of its hazardousness or contamination [14,15], relating darker colors to dusts more contaminated by heavy metals, which could provide a quick and economical “proxy” method of diagnosis, although there are studies where these results have not been so evident [16]. Magnetism is another property that has been used by some authors to estimate the degree of contamination of soils [17,18]. The application of magnetic methods can be useful to study areas affected by contaminants, as they are relatively faster than chemical analysis methods. They are based on the knowledge of the concentration of magnetic particles and magnetization-inducing minerals, determining the concentration of anthropogenic ferrimagnetic minerals in soil or urban dust samples. Metals with magnetic properties are iron (Fe), cobalt (Co), and nickel (Ni). Ferrimagnetic minerals are carriers of heavy metal ions, so if urban dust contains ferrimagnetic minerals, it is likely to contain heavy metals as well [19,20].

All of the above does not remove the need to carry out a chemical analysis in which the concentration of each heavy metal is determined to evaluate the level of contamination of the samples and then apply geoenvironmental indices that allow us to evaluate the level of contamination and health indices [15,16,21], in order to know the risk to which the population is exposed and be able to adopt the appropriate measures.

In this paper, we want to highlight the presence of heavy metals in schoolyard dust and the quantity they are found. We investigated the hypotheses that (1) there is a relationship between the dark color and higher degree of magnetism of urban dust and the contamination of dust collected in schoolyards, and (2) schoolyards with a higher concentration of magnetic materials are those with a higher concentration of heavy metals. Therefore, we aimed to demonstrate whether schoolyards are safe for children from an environmental and health point of view.

The objectives set out to address our hypotheses were as follows:

– To determine the presence of heavy metals, as well as the color and degree of magnetism, of urban dust sampled from schoolyards in the city of Murcia, relating color to the degree of magnetism;
– To evaluate the concentrations of heavy metals in the dust samples, study the possible relationships between them, and characterize the magnetic fraction of urban dust;
– To relate the color and degree of magnetism with the load of toxic pollutants in urban dust;
– To apply different geoenvironmental and health indices to determine the degree of environmental hazard in schools and its possible direct impact on children’s health.

## 2. Materials and Methods

**Figure 1:**
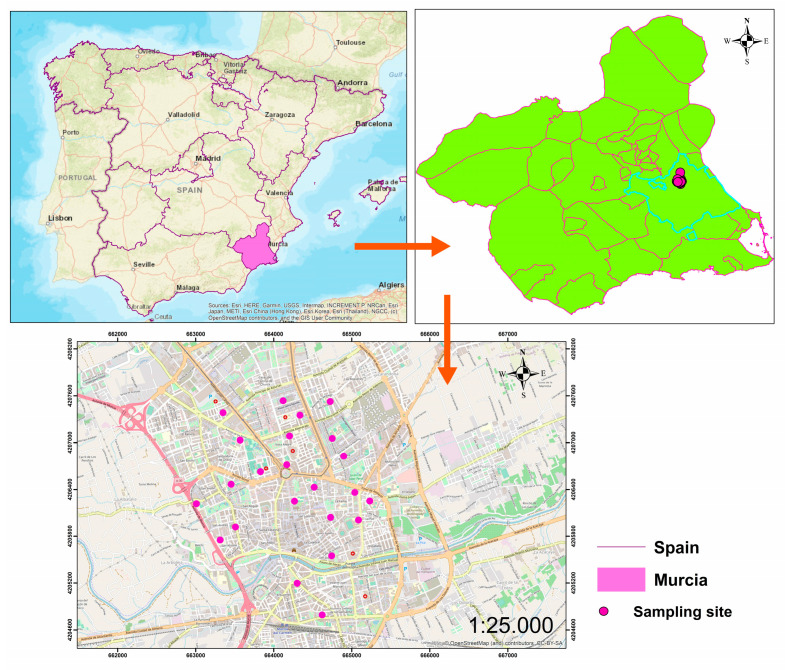
The location of the study area and sampling sites.

Sampling was carried out in 27 schools in the center of the city of Murcia (Figure 1) between February and November 2023, coinciding with a period of low rainfall (Table S1). Murcia is a city located in Southeastern Spain, characterized by a semi-arid Mediterranean climate, with an average annual rainfall of 300 mm and an average annual temperature of 18 °C. The dust samples were collected on the sports field and in the children’s play area of each school; therefore, in most of the schools, two samples were taken. Each of these samples was the result of mixing three subsamples. For dust sampling, a total area of 3 m^2^ (1 m^2^ for each subsample) was brushed, and the collected dust was stored in an airtight plastic container. The collected dust samples were sieved at 1 mm to discard any plant debris or other artifacts. The samples were then stored in a dry place at 4 °C until analysis.

### 2.1. Sample Analysis

To determine the composition of urban dust, the samples were ground in an agate mortar and mounted on a petrographic slide for analysis via X-ray diffractogram. The Philips equipment has a sensitivity of 5 × 10^3^ and a vertical goniometer. The working conditions were Kα Cu radiation; intensity of 24 mA; 40 kV; Ni filter; window slit of 1; meter gap of 0.1; and scanning speed of 1°_ 2θ min^−1^.

The color of the samples was determined with Munsell keys [22]; thus, the samples were grouped into two categories according to their value: light, with a value between 5 and 6, and dark, with a value between 2 and 4.

To determine the magnetism of the urban dust samples, a 3 cm diameter neodymium magnet, capable of attracting magnetic particles (ferri, ferro, and paramagnetic), was used. The magnet was placed over the dust, and the particles adhering to it were removed with a fine brush. Depending on the weight of the particles attracted by the magnet, three categories of magnetism were determined: high (>30%), medium (15–30%), and low (<15%).

The samples, prior to acid digestion, were ground using an agate mortar and passed through a 50 µm sieve. Acid digestion was carried out with aqua regia (HNO_3_/HCl, 1:3) in a microwave oven at 220 °C for 1 h. The analysis of the samples was performed by means of ICP-OES, using a Thermo ICAP 6500DUO, (Waltham, MA, USA) to determine the total concentrations of cadmium (Cd), copper (Cu), chromium (Cr), nickel (Ni), lead (Pb), and zinc (Zn).

A certified soil (SRM San Joaquin) was used as a reference to calculate the recoveries of the selected elements Cd (95–101), Cu (92–98), Zn (94–102), Cr (95–99), Ni (98–102), and Pb (92–98%). The samples were analyzed in triplicate, and a standard deviation (2–4%) was obtained.

The magnetic and non-magnetic fractions separated with the magnet were analyzed using the same ICP-OES methodology described above, and the same heavy metals were determined. We observed the morphology via scanning electron microscopy (SEM) with an energy-dispersive system (EDS).

### 2.2. Environmental Pollution Indices and Potential Risk

The equations used to calculate the environmental and health indices are shown in Table 1 and Table 2. Table 1 shows all the geochemical indices with their Equations (1)–(6), while Table 2 shows the health-related indices Equations (7)–(12). These indices have been used in other studies by the authors of this study [21]. The total pollutant load (TPL) is the sum of the concentrations of all the elements studied.

**Table 1:** Environmental pollution indices, their equations, and levels of contamination (interpretation of each index).

Index	Equation	Interpretation of Index
Contamination factor (CF)	$\mathrm{CF}=\frac{\mathrm{Cn}}{\mathrm{Cbn}}$ (1)	Cn: heavy metal; Cbn: background value (mg kg^−1^) CF < 1 (low), 1 ≤ CF ≤ 3 (moderate), 3 ≤ CF < 6 (considerable), CF ≥ 6 (very high)
Degree of contamination (Cdeg)	Cdeg=CF1+…CFn (2)	Cdeg < 8 (low), 8 ≤ Cdeg < 16 (moderate), 16 ≤ Cdeg < 32 (considerable), 32 ≥ Cdeg (very high)
Pollutant load index (PLI)	$\mathrm{PLI}={\left(\mathrm{CFMe}1\times \mathrm{CFMe}2\times \ldots \mathrm{CFMen}\right)}^{\frac{1}{n}}$ (3)	Me: metal PLI < 1 (absence) PLI > 1 (presence of heavy metal contamination)
Enrichment factor (EF)	$\mathrm{EF}=\frac{\left(\frac{\mathrm{Cx}}{\mathrm{Cref}}\right)\mathrm{sample}}{\left(\frac{\mathrm{Cx}}{\mathrm{Cref}}\right)\mathrm{background}}$ (4)	Cx =metal element (mg kg^−1^); Cref = reference element (mg kg^−1^)—in our case, Ca+2; EF < 2 (minimal), EF = 2–5 (moderate), EF = 5–20 (substantial), EF = 20–40 (very high), EF > 40 (extremely high)
Geo-accumulation index (Igeo)	$\mathrm{Igeo}=\log2\frac{\mathrm{Cn}}{K\times \mathrm{Cbn}}$ (5)	Factor K = 1.5 Igeo < 0 (uncontaminated), 0–1 (uncontaminated–moderate), 1–2 (moderate), 2–3 (moderate–high), 3–4 (high), 4–5 (high–very high), >5 (very high)
Potential ecological risk index (RI)	RI = ∑ErMe (6) ErMe = TrMe × CFMe	TrMe = toxic response factor for each metal (Me), CFMe = contamination factor for each metal RI < 150 (low), 150 ≤ RI < 300 (moderate), 300 ≤ RI < 600 (considerable), RI ≥ 600 (very high)

**Table 2:** Health-related indices and cancer risk (CR), their equations, and levels of contamination (interpretation of each index).

Index	Equation	Interpretation of Index
Ingestion (Ding)	$\mathrm{Ding}=\frac{C\times \mathrm{ingR}\times \mathrm{EF}\times \mathrm{ED}}{\mathrm{Bw}\times \mathrm{AT}}\times 10^{-6}$ (7)	IngR, the intake rate (mg day^−1^), is assumed to be 100 for adults and 200 for children; C is the content of toxic metals (mg kg^−1^); EF is the exposure factor used in the present study (days year^−1^), which is 365; ED is the exposure period (years), assumed in the present study to be 6 for children and 24 for adults; AT is the average time contact, defined as ED × 365 days for non-carcinogens and 70 × 365 = 25,550 days for carcinogens; and Bw is the mean body weight (kg), defined as 15 for children and 70 for adults.
Inhalation (Dinh)	$\mathrm{Dinh}=\frac{C\times \mathrm{inhR}\times \mathrm{EF}\times \mathrm{ED}}{\mathrm{PEF}\times \mathrm{Bw}\times \mathrm{AT}}$ (8)	InhR, the inhalation rate (m^3^ day^−1^), is estimated to be 7.6 for children and 20 for adults. PEF is the particulate emission factor (m^3^ kg^−1^), which is 1.36 × 10^9^. The other factors have been explained in the equation above.
Dermal contact (Dderm)	$\mathrm{Dderm}=\frac{C\times \mathrm{SL}\times \mathrm{SA}\times \mathrm{ABS}\times \mathrm{EF}\times \mathrm{ED}}{\mathrm{BW}\times \mathrm{AT}}\times 10^{-6}$ (9)	Dderm is the dermal contact rate, where SL is the skin level (mg cm^−2^ day^−1^), which, in this study, was taken as 0.2 for children and 0.07 for adults; SA is the exposed skin area (cm^2^), estimated to be 2800 for children and 5700 for adults; ABS is the skin absorption factor, considered as 0.001 for all the heavy metals studied. The other factors have been explained in the equation above.
Lifetime median daily dose (LADDinh)	$LADDinh=\frac{C\times EF}{AT\times PEF}\times \left(\frac{inhRchild\times EDchild}{Bwchild}+\frac{inhRadult\times EDadult}{Bwadult}\right)$ (10)	
Hazard index (HI)	HI = HQing + HQinh + HQder (11)	The HQs for each exposure route were calculated by dividing the mean daily dose from each exposure route (Ding, Dinh, and Dder) by a corresponding reference dose (RfDing, RfDinh, and RfDder) (Table 3). The RfD values (mg kg^−1^ day^−1^) illustrate the maximum permissible risk in the daily exposure of citizens throughout their lives. When HI < 1, it shows that there are no adverse health effects; when HI > 1, non-cancer health impacts may occur
Cancer risk (CR)	$\mathrm{Cancer}\;\mathrm{Risk}\;\left(\mathrm{CR}\right)=\mathrm{LADDinh}\times \mathrm{SF}$ (12)	SF: slope factor. Cd: 6.30 × 10^0^; Cr: 4.20 × 10^1^; Ni: 8.40 × 10^−1^; Pb: 4.20 × 10^−2^.Risk management decisions should be made when CR ranges from 10^−6^ to 10^−4^.

**Table 3:** Reference doses (mg kg^−1^ day^−1^) (RfDing, RfDder, and RfDinh).

	Cd	Cr	Cu	Ni	Pb	Zn
RfDing	1.00 × 10^−3^	3.00 × 10^−3^	4.00 × 10^−2^	2.00 × 10^−2^	3.50 × 10^−3^	3.00 × 10^−1^
RfDderm	1.00 × 10^−5^	6.00 × 10^−5^	1.20 × 10^−2^	5.40 × 10^−3^	5.25 × 10^−4^	6.00 × 10^−2^
RfDinh	1.00 × 10^−3^	2.86 × 10^−5^	4.02 × 10^−2^	2.06 × 10^−2^	3.52 × 10^−3^	3.00 × 10^−1^

The background surface of our research was asphalt, as it was the most common surface on which the sampled dust samples were deposited. The average values measured on asphalt were as follows: Cd: 0.3 mg kg^−1^; Cr: 13.2 mg kg^−^^1^, Cu: 18.1 mg kg^−^^1^; Ni: 11.3 mg kg^−^^1^; Pb: 4.3 mg kg^−^^1^; and Zn: 34.5 mg kg^−^^1^. The reference element (Cref), chosen for its abundance in SE Spain, was Ca^+2^, whose value was 26.5 (g kg^−^^1^). The asphalt analysis was carried out with the same methodology as the dust samples.

### 2.3. Statistical Analysis

General statistical analyses were performed, and frequency tables relating the categorical variables of color and magnetism were produced using the statistical program Jamovi program (Version 0.9), Computer Software 2018 [23]. Correlation analysis (Spearman’s rank coefficients), principal component analysis, and cluster analysis (dendrogram constructed via Ward’s method) was carried out using the software package IBM SPSS 28.0 for Windows.

## 3. Results

**Figure 2:**
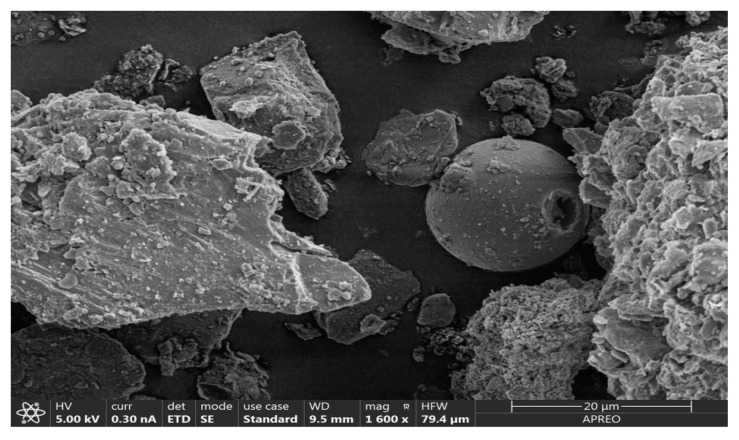
SEM image of sampled urban dust. Magnification is 1600×.

The matrix of urban dust was similar in all samples and consisted of a rather heterogeneous mixture, dominated by calcite (45–50%), dolomite (25–30%), quartz (less than 20%), and gypsum, with phyllosilicates representing a small percentage (<5). The presence of traces of magnetite and other iron oxides (hematite, magnetite, and goethite) could be observed, as can be seen in the electron microscopic image (Figure 2), which appear as spheres with a smooth surface. Smooth spherical particles are generated during combustion processes. They arise from the melting of impurities in a fuel material and take this shape due to surface tension [24].

The concentrations of the six heavy metals analyzed varied greatly depending on the element in question and the sample analyzed, as can be seen in Table 4, where their mean, maximum, minimum, and standard deviation (SD) values are shown.

According to these values, the order of abundance of these heavy metals in the urban dust samples was Zn > Cu > Cr > Pb > Ni > Cd.

An investigation was carried out to determine the possible relationships between the selected heavy metals. The principal component analysis, as can be seen in Table 5, revealed two groups: (1) the first group formed by Cd, Cu, Ni, and Zn, which explains 44.7% of the variance, and (2) the second group formed by Cr and Pb, which explains 26.8% of the variance.

Table 6 shows the correlations found between the elements under study, with the highest levels of significance. The correlation between Ni and Cu stands out for its value (0.822, *p* < 0.01). In the case of Pb, its correlations with other elements are not statistically significant.

**Figure 3:**
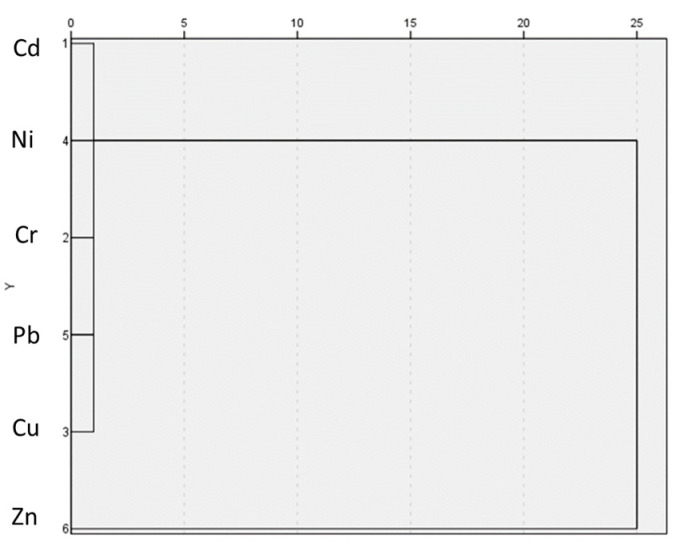
Dendogram with distribution of elements.

As can be seen in Figure 3, all the elements, except Zn, form a more homogeneous group.

In terms of color, all samples had the same hue of 2.5 Y. According to the results obtained, slightly more than half of the samples had a dark color. Table 7 shows the results obtained by comparing the color of the samples with their degree of magnetism.

Regarding the relationship between the color and degree of magnetism of the samples and the total pollutant load (TPL), the results obtained are presented in Table 8.

The determination of magnetism showed that most of the samples had a medium (15 to 30%) or low (<15%) content of magnetic particles, with only five samples containing more than 30% of magnetic particles.

**Figure 4:**
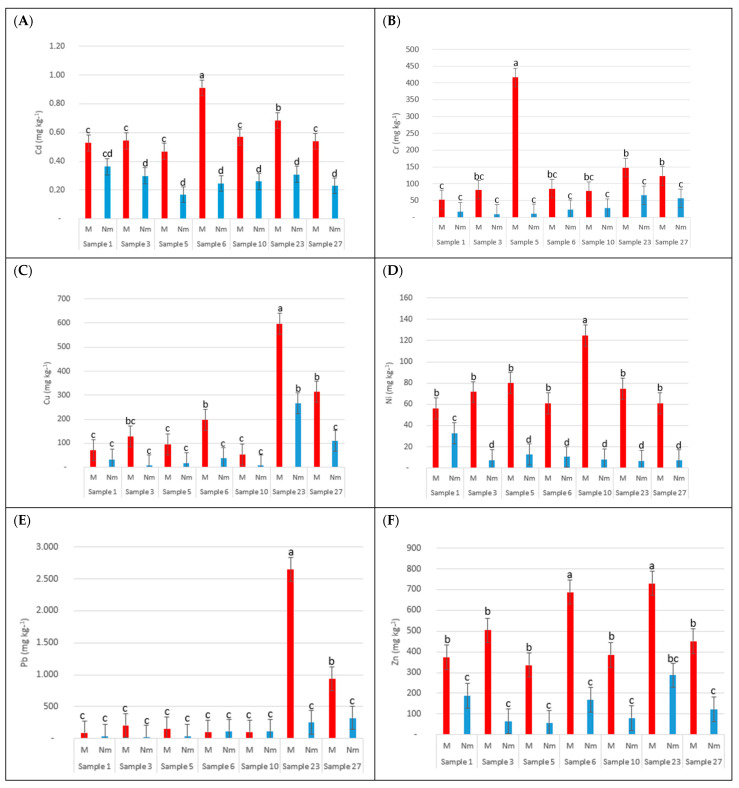
Concentrations of the magnetic (M) and non-magnetic (Nm) fractions of different elements in some samples: (**A**) (Cd), (**B**) (Cr), (**C**) (Cu), (**D**) (Ni), (**E**) (Pb), and (**F**) (Zn). Different letters indicate significant differences between the M and Nm fractions in each of the samples for each element at the 95% confidence level.

Figure 4 clearly shows higher concentrations of Cd, Ni, and Zn in the magnetic fraction (M) of the powder samples in relation to the non-magnetic fraction (Nm), with statistically significant differences. However, for Cr, Cu, and Pb, higher trends are observed in M than in Nm, but without statistically significant differences in all samples. On the other hand, and from a quantitative point of view, the difference in concentrations between the fractions was not equal for all heavy metals; for Cd, an average concentration of 0.6 and 0.3 mg kg^−1^ was found in the magnetic and non-magnetic fractions, respectively, while for Ni, it was 76 and 12 mg kg^−1^, respectively, showing an average concentration that was more than 6 times higher in M than in NM. These results suggest that there is a positive correlation between the magnetism of dust and the heavy metals present in it, but their affinities for this property are very different.

### 3.1. Environmental Pollution Indices

These indices were used to assess the environmental risk arising from exposure to heavy metals in dust in schools where children carry out their daily activities. They are a very useful tool to assess the current situation so that action can be taken in case of risk [25]. The values obtained are presented in Table 9.

As for the global indices of environmental pollution, the degree of pollution (Cdeg) had a value of 38.8 in this study, the index of potential ecological risk (RI) was calculated to be 178.05, and the pollutant load index (PLI) had a value of 3.79, which shows the presence of heavy metal contamination when it is higher than 1.

In the CF, EF, and Igeo indices, Zn and Pb are the elements with the highest values, indicating high contamination.

**Figure 5:**
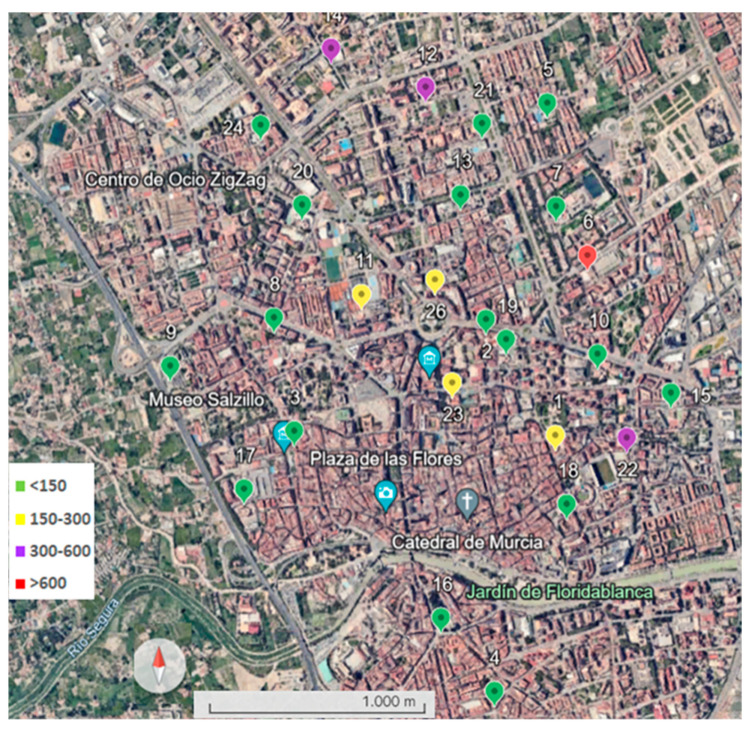
Spatial diversity of ecological risk (IR). Green color represents low ecological risk with values in the interval RI < 150; yellow color shows moderate ecological risk in the interval 150 ≤ RI < 300; purple color shows considerable ecological risk in the interval 300 ≤ RI < 600; and red color represents very high ecological risk with RI ≥ 600.

The potential ecological risk index (RI) is spatially represented in Figure 5.

The points outside the map correspond to specific sampling sites 27 (green) and 25 (yellow) with low and moderate ecological risk, respectively.

### 3.2. Human Health Indices (HI) and Cancer Risk (CR)

The values obtained for different health indices for both adults and children are presented in Table 10.

Regarding cancer risk (CR), Cr, Cd, Ni, and Pb were assessed using lifetime inhalation exposure. The results obtained are presented in Table 11.

## 4. Discussion

The composition of the urban dust found in schools in Murcia is like that found by other authors in schoolyards [26,27]. Perhaps what differs most is the concentrations of the heavy metals analyzed. In our study, the most abundant metals in the dust samples were in the order of Zn, Cu, Cr, and Pb, with Ni and Cd being less abundant. The values of these heavy metals were all lower than those found by Marín-Sanleandro et al. [16] in urban dust sampled on the streets of Murcia, which could be due to the greater cleanliness inside these schools and the presence of vegetation or construction barriers that delimit their areas, thereby preventing the passage of these pollutants from nearby traffic roads [7].

The highest concentration in samples shows Zn, similar to what was found in the dusts sampled from other schools or cities, as reported in a study in Malaysia [28] and in a systematic review of schools by Moghtaderi et al. [7,29]. Although these studies were carried out by taking samples inside classrooms, we can assume that what is found inside classrooms reflects what is found outside [30].

Cu is an essential trace element for humans, acting as a necessary cofactor for many enzymes and proteins. However, its excess can be toxic due to its high oxidative capacity. Its presence in schoolyards can arise from many different causes, as it can be present in plastic and rubber materials, paints and varnishes, printing inks, synthetic fibers, and metallic coatings, etc., all of which are commonly found in schools [31].

The fact that the amount of Cr exceeded that of Pb in the schoolyards might be due to paint residues from sports fields and other components of schoolyards. The presence of Cr has been associated with the use of paint or steel welding [32].

Based on the work by Zgłobicki et al. (2018) [33], but with some differences, in our work, two groups of elements could be determined from the principal component analysis; the first group (Zn, Ni, Cu, and Cd) is related to the mixed effects of natural factors such as lithologies from nearby areas, together with anthropogenic activities, such as the effect of vehicles, and the second group (Cr and Pb) is related more specifically to anthropogenic activities (Table 5). The fact that Zn is separated into another group in the cluster analysis may indicate a different origin from the rest of the elements that form a more homogeneous group (Cd, Ni, Cr, Pb, and Cu) (Figure 3).

All the sampled schools, located in the urban center of the city, are near roads, and some are close to highways, so we cannot rule out that the origin of the heavy metals found in the dust of their schoolyards may be related to road traffic. Similar results have been reported in other studies [8,27,34,35].

With regard to the relationship between the color of the samples and the content of magnetic particles (Table 7), all samples with a high content of magnetic particles were dark in color. Samples with light colors generally had a low degree of magnetism, although we also detected dark-colored samples with a medium or even low degree of magnetism. However, this does not mean that magnetism and pollution are not related since the results obtained from comparing color and magnetism with the total pollutant load (TPL; Table 8), calculated as the sum of the concentrations of pollutant elements in urban dust such as Cd, Cr, Cu, Ni, Pb, and Zn, show that there is a statistically significant relationship between these parameters. The highest concentration of pollutants was found in dark-colored dust samples with medium or high magnetism, which confirms our hypothesis.

These magnetic particles must have an anthropogenic origin and come from the combustion of fossil fuels used in industries and motor vehicles. The magnetism present in these particles could be due to the retention of metallic elements such as Fe or Ni [36], or the presence of metals in the form of oxides [37]. The study of the composition of this fraction via ICP-MS revealed the concentration of heavy metals associated with it, with very high values in the case of Pb, suggesting that most of the contamination present in dust would be concentrated in the magnetic fraction. This could be useful in the case of having to take urgent decontamination measures.

Considering the affinity of heavy metals for the magnetic fraction of urban dust [38], it would be advisable to develop techniques that make use of this property to decontaminate urban dust, especially in street cleaning machinery, where, by installing appropriate devices, heavy metals could be concentrated in the magnetic fraction, which usually represents at most 20% of the total, and then managed as hazardous waste.

Regarding the results obtained for the environmental pollution indices, the values of the contamination factor (CF), which describes the level of dust pollution for a given element, indicate that the level of pollution is moderate for Cd and Ni, considerable for Cr and Cu, and very high in the case of Pb and Zn (values higher than 6), values similar to our results were obtained by Aguilera et al. 2022 in several Mexican cities [39].

The EF indicates the contamination of urban dust by an element in relation to its background value—in this case, asphalt—and a common and abundant reference element in the study area—in this case, Ca^2+^. Cd exhibited minimal enrichment, Cu and Ni exhibited moderate enrichment, and Cr, Zn, and Pb exhibited substantial enrichment, with the highest values obtained for Zn and Pb (18.11 and 19.09, respectively). For dusts sampled from the streets of an urban area in Murcia [16], the value was 45 for Pb, which was more than double the value found in schools, and 20 in the case of Zn, which was very similar to that obtained in this work. In their study of some kindergartens in the same city where children also spend a lot of time daily, Moltó found higher enrichment factor values for Cd, Cr, Cu, and Ni, but lower values for Zn and Pb [40].

The values of the Igeo, which shows the level of contamination in the schoolyards, indicated no or very low contamination by Cd (0.67), Cu (0.92), and Ni (0.13). Moderate contamination was found for Cr (1.40) and Pb (2.25), and only Zn (2.53) had values that could be interpreted as moderate to high contamination. The values of this index are highly variable depending on the matrix studied (sediments, soil, dusts) and the geographical location sampled [33,41].

There are some global indices of environmental pollution that express contamination by heavy metals in general without differentiating between the elements. One of these is the degree of contamination (Cdeg). In this study, its value was 38.8, which implies, according to the scale used for this index, a very high degree of environmental contamination in the schools. Other authors who used Cdeg have also found very high values in their research [42], and it seems to be an environmental index that is very sensitive to heavy metal pollution.

In regard to the potential ecological risk index (RI), which is the sum of the potential risk of each of the heavy elements considering their toxicity, the value obtained (165.42) corresponded to moderate ecological risk, although the spatial domain corresponded to low values. The highest value of this index was 674 (high potential ecological risk) (Figure 5). The average RI value was five times lower than the value found in other parts of the region, such as the city of Cartagena [43].

The value obtained for the pollutant load index (PLI), a global index that considers the CF of all heavy metals, was 3.79, which indicates the presence of contamination by heavy metals as it was greater than 1. This PLI value was also below that found in other parts of the region [43].

As can be seen from the results, depending on the sensitivity of the global index used, the risk or degree of environmental contamination is more or less high, but in all cases, contamination by heavy metals is present in the school environment.

To assess the health risk caused by exposure to heavy metals in dust in schools where children, teachers, and other staff carry out their daily activities, health indices were examined. These indices are a very useful tool that reveals the current situation, enabling steps to be taken in case of risk. According to the results presented in Table 10, for non-carcinogenic effects, ingestion is the main route of exposure and entry of dust particles into the body, which poses the main health risk for adults and children for all heavy metals. The second route is dermal contact, and inhalation is the route with the lowest values. The HQing and HQderm values are between 100 and 10,000 times greater than the corresponding HQinh values; these results coincide with those found in other works [21,30,44,45,46,47] that assessed health risks in other locations.

As ingestion and dermal contact are the main routes of entry of heavy metals at school sites, the cleanliness of playgrounds and places where children play and come into direct contact with objects on the ground should be maximized.

The HI values for dust range from 0.0054 for Cd to 0.29 for Cr in children and from 0.00076 for Cd to 0.036 for Cr in adults. The HI values decrease in the order Cr > Cu > Zn > Pb > Ni > Cd in children, while in adults, the order is Cr > Pb > Cu > Zn > Ni > Cd. All HI values for all elements and for both adults and children are less than 1, indicating that there is no risk of developing adverse health effects. In a study that took place at a Murcia garden, the HI values for children were lower for Cd, Cr, Cu, and Ni and again higher for Pb and Zn [26].

Regarding cancer risk (CR), Cu, Cr, Ni, and Pb were assessed using lifetime inhalation exposure. As can be seen in Table 10, the CR values in urban dust decrease in the order of Cr > Ni > Cd > Pb.

Authorities should make decisions and implement measures when the cancer risk index is between 10^−6^ and 10^−4^ [21]. The values in the present study are well below this range, so the current situation at schools is not considered a risk and should not cause alarm, although it should be borne in mind that Cr has been found to be the most toxic element with the highest risk to health, so preventive measures should be taken. The Cr found in schoolyards may come from the paints and enamels used on different surfaces and objects in the playground, as anti-corrosive and galvanized products are used in their manufacture [47,48]. Roughly speaking, the paints have been manufactured with hexavalent chromium, while the trivalent, which is inert, comes from the corrosion of steel. However, other sources, such as road traffic, cannot be ruled out, especially since all the schools in this study are located in an urban area of the city, as the wear and tear of vehicle brakes, wheels, and engines can be sources of Cu, Cr, Zn, and Pb pollutants, and gases and particles are produced during combustion [15].

To prevent the entry of particulate pollutants, the presence of vegetation barriers may be highly recommended in schools as it would reduce their entry and, thus, the exposure of children to air pollution [49,50]. In addition, the presence of vegetation in schools is associated with several benefits, ranging from improved academic performance to the promotion of children’s mental and physical health. Many studies have demonstrated the negative influence of environmental pollution in schools on children’s cognitive development [51,52,53]; therefore, the cleanliness of schoolyards should be maximized, with the entry of polluting particles being prevented as much as possible.

## 5. Conclusions

Zn was the most abundant heavy metal found in the dust samples obtained from the schools, followed by Cu and Cr.

In view of the values of the environmental pollution indices, Pb and Zn are the heavy metals that present the greatest environmental pollution problems. Likewise, the general environmental pollution indices showed that the existing ecological risk is moderate; however, the index that measured the degree of pollution by heavy metals (Cdeg) was very high.

Regarding the health indices, ingestion is the most important route of exposure to heavy metals in both children and adults. The health index values for children were higher than those for adults, confirming that heavy metals in school dust represent a potential health risk for children. Cr is the most toxic element with the highest risk to children’s health. This element could be related to the presence of painted objects and steel welds.

The assessment of cancer risk shows there is no risk for children or adults in the schools.

The concentration of contaminating elements was highest in the dusts that had a dark color and medium or high magnetism. An analysis of the magnetic and non-magnetic fractions indicated appreciable concentrations of all heavy metals in the magnetic fraction.

Schools should be safe places for children. Therefore, authorities should be aware that in schools in Murcia, although there is no risk of cancer for children or adults, there is an ecological risk due to the presence of heavy metals in schoolyards. Hence, it is recommended that attention is paid to all building materials used in these places; frequent cleaning and washing of schoolyards are implemented to prevent the accumulation of dust; and the number of green barriers or construction barriers is increased in schools to prevent the entry of pollutants as much as possible. We believe it is necessary to conduct a similar study in rural schoolyards in our region, in areas away from pollution, to characterize the dusts in these schoolyards.

## Figures and Tables

**Table 4:** Concentrations of heavy metals (mg kg^−1^) and TPL (Total Pollution Load) in urban dusts sampled from schools in the city of Murcia.

	Cd	Cr	Cu	Ni	Pb	Zn	TPL
Mean	0.4	68	77	19	56	454	674
Median	0.3	49	39	16	23	266	392
Maximum	8.2	256	514	81	385	3519	4761
Minimum	0.1	13	9	4	5	53	84
CV	269	75	115	72	149	132	96

**Table 5:** Principal component analysis.

	Factor 1	Factor 2
Cd	**0.828**	−0.039
Cr	0.200	**0.881**
Cu	**0.903**	0.209
Ni	**0.868**	0.224
Pb	−0.068	**0.857**
Zn	**0.510**	−0.057
Variation percentage (%)	44.7	26.8
Total variance (%)		69.5

Note: Bolded values indicate statistically the most important impact.

**Table 6:** Bivariate correlation.

	Cd	Cr	Cu	Ni	Pb	Zn
Cd	1	0.283	**0.534**	**0.638**	0.254	**0.579**
Cr		1	**0.556**	**0.568**	0.373	0.360
Cu			1	**0.822**	0.106	**0.687**
Ni				1	0.096	**0.577**
Pb					1	0.205
Zn						1

Note: Bolded values indicate the correlation is significant at the 0.01 level.

**Table 7:** Relationship between the sample color and percentage of magnetic particles.

		Color	
Magnetic Particles (%)		Dark	Light
Low (<15)		7	15
Medium (15–30)		17	6
High (>30)		5	0
	**Value**	***p***	
χ^2^	12.2	0.001	

**Table 8:** Relationship between the color and degree of magnetism of the samples and the total pollutant load (TPL).

	Color	
Magnetic Particles (%)	Dark	Light
Low (<15)	4229	7810
Medium (15–30)	12,325	4133
High (>30)	5194	0
	**Value**	***p***
χ^2^	8175	<0.001

**Table 9:** Average values of the environmental pollution indices. CF, contamination factor; EF, enrichment factor; Igeo, geo-accumulation index.

	Cd	Cr	Cu	Ni	Pb	Zn
CF	1.57	5.16	4.28	1.65	13.05	13.15
EF	1.68	7.11	5.74	2.13	19.09	18.11
Igeo	0.67	1.40	0.92	0.13	2.25	2.53

**Table 10:** Mean non-carcinogenic index values for children and adults: Ding, Dinh, Dderm (mg kg^−^^1^ day^−^^1^), and hazard quotient for each element and route of exposure.

Children							
	Ding	Dinh	Dderm	HQing	HQinh	HQderm	HI
Cd	5.4 × 10^−6^	1.5 × 10^−10^	8.7 × 10^−9^	5.4 × 10^−3^	1.5 × 10^−7^	1.5 × 10^−5^	5.4 × 10^−3^
Cr	8.7 × 10^−4^	2.4 × 10^−8^	1.4 × 10^−6^	2.9 × 10^−1^	8.5 × 10^−4^	4.1 × 10^−4^	2.9 × 10^−1^
Cu	9.9 × 10^−4^	2.8 × 10^−8^	1.6 × 10^−6^	2.5 × 10^−2^	6.9 × 10^−7^	2.3 × 10^−6^	2.5 × 10^−2^
Ni	2.4 × 10^−4^	6.7 × 10^−9^	3.8 × 10^−7^	1.2 × 10^−2^	3.2 × 10^−7^	1.2 × 10^−6^	1.2 × 10^−2^
Pb	6.1 × 10^−5^	2.0 × 10^−8^	1.1 × 10^−6^	1.7 × 10^−2^	5.7 × 10^−6^	3.8 × 10^−5^	1.8 × 10^−2^
Zn	5.8 × 10^−2^	1.6 × 10^−7^	9.3 × 10^−5^	1.9 × 10^−2^	5.4 × 10^−7^	2.7 × 10^−6^	1.9 × 10^−2^
**Adults**							
	**Ding**	**Dinh**	**Dderm**	**HQing**	**HQinh**	**HQderm**	**HI**
Cd	5.8 × 10^−7^	5.5 × 10^−11^	1.8 × 10^−9^	5.8 × 10^−4^	5.5 × 10^−8^	1.8 × 10^−4^	7.6 × 10^−4^
Cr	9.3 × 10^−5^	8.8 × 10^−9^	2.8 × 10^−7^	3.1 × 10^−2^	3.1 × 10^−4^	4.7 × 10^−3^	3.6 × 10^−2^
Cu	1.1 × 10^−4^	1.0 × 10^−8^	3.2 × 10^−7^	2.7 × 10^−3^	2.5 × 10^−7^	2.7 × 10^−5^	2.7 × 10^−3^
Ni	2.5 × 10^−5^	2.4 × 10^−9^	7.7 × 10^−8^	1.3 × 10^−3^	1.2 × 10^−7^	1.4 × 10^−5^	1.3 × 10^−3^
Pb	7.6 × 10^−5^	7.2 × 10^−9^	2.3 × 10^−7^	2.2 × 10^−2^	2.0 × 10^−6^	4.4 × 10^−4^	2.2 × 10^−2^
Zn	6.2 × 10^−4^	5.8 × 10^−8^	1.9 × 10^−6^	2.1 × 10^−3^	1.9 × 10^−7^	3.2 × 10^−5^	2.1 × 10^−3^

**Table 11:** Average cancer risk values for Cd, Cr, Ni, and Pb.

Cd*LADD*	Cr*LADD*	Ni*LADD*	Pb*LADD*	Cd*CR*	Cr*CR*	Ni*CR*	Pb*CR*
3.2 × 10^−11^	5.1 × 10^−9^	1.4 × 10^−9^	4.2 × 10^−9^	2.0 × 10^−10^	2.1 × 10^−7^	1.2 × 10^−9^	1.8 × 10^−10^

## Data Availability

All data are contained within the article.

## Supplementary Materials

The following are available online at https://www.mdpi.com/article/10.3390/toxics12110804/s1, Table S1: Description of some characteristics of the schools and of the dust samples sampled.
