# Supplementary material for: Geoenvironmental and Health Indices to Assess the Hazardousness of Heavy Metals in Urban Dust in Schoolyards in Murcia, Spain
Source: Toxics. 2024 Nov 7;12(11):804. doi: 10.3390/toxics12110804 (PMC11598568; doi:10.3390/toxics12110804)
Supplement: Supplementary file 1 [file toxics-12-00804-s001.zip › toxics-3256358-supplementary.pdf]

Table S1: Description of some characteristics of the schools and of the dust samples sampled

Schools	Sample color (Munsell Key)	Magnetism %	Traffic intensity Vehicles/week	Nº of childrens	Nº of adults
1	2.5Y 4/3	<15	<45,000	450	31
2	7.5YR5/1	<15	>65,000	675	55
3	Gley2 4.5/10B	<15	45,000-65,000	312	34
4	10YR 4/5	<15	45,000-65,000	512	43
5	2.5YR 4/6	<15	<45,000	275	35
6	7.5YR 5/3	<15	>65,000	180	19
7	2.5YR 4/4	<15	>65,000	252	16
8	10YR 3.5/3	<15	45,000-65,000	225	20
9	2.5Y 3/3	<15	>65,000	225	20
10	7.5YR 3/4	15-30	>65,000	675	55
11	10YR 3/2	15-30	>65,000	410	27
12	2.5Y 4/4	<15	<45,000	413	44
13	2.5Y 4/3	<15	>65,000	675	55
14	2.5Y 3/2	<15	>65,000	675	55
15	10YR 3/1	<15%	45,000-65,000	525	42
16	2.5Y 4/2	>30	>65,000	675	40
17	7.5YR 6/2	15-30	>65,000	153	20
18	10YR 5/4	15-30	45,000-65,000	90	14
19	10YR 6/2	<15	>65,000	396	45
20	5Y 5/2	>30	45,000-65,000	211	18
21	2.5Y 5/2	>30	>65,000	225	20
22	7.5YR 5/3	<15	<45,000	378	30
23	10YR 6/4	<15	>65,000	450	40
24	10YR4/3	<15	>65,000	450	40
25	10YR 6/3	15-30	45,000-65,000	225	15
26	2.5Y 4/3	15-30	>65,000	450	40
27	7.5YR5/1	<15	<45,000	450	40
